# Obtaining PDC and other high-added value products from lignin by *in silico* genetic engineering in *Novosphingobium aromaticivorans*

**DOI:** 10.1515/jib-2024-0059

**Published:** 2026-02-25

**Authors:** Isabel María Fernández, Francisco Guil, José Manuel García

**Affiliations:** Faculty of Biology, University of Murcia, CEIR Campus Mare Nostrum, Campus de Espinardo, 30100 Murcia, Spain; Faculty of Computer Science, University of Murcia, CEIR Campus Mare Nostrum, Campus de Espinardo, 30100 Murcia, Spain

**Keywords:** metabolic models, genetic interventions, coupled growth, lignin, PDC production

## Abstract

Lignin, the second most abundant plant biopolymer on Earth, is produced in large quantities as waste material by many industries. Researchers have studied bacterial metabolic networks as potential candidates for integrating lignin into a biotechnological value chain. The GEM used in this work for metabolic engineering is iNovo479, which simulates the metabolism of *Novosphingobium aromaticivorans* DSM12444. We have conducted a study on PDC production and found several intervention strategies to help achieve this goal. These strategies include more than just blocking the *ligI* gene, which has been a well-known approach. Although these new strategies resulted in a lower yield of PDC relative to biomass formed, they led to a higher cell yield than deleting the *ligI* gene. The research presented in this paper focuses on the production of high-value compounds from lignin. Previous studies have used mutated microorganisms to produce these bioproducts from large amounts of glucose. However, biosynthesis from lignin would improve productivity and make the fermentation process more cost-effective. Through gene knockouts, we have discovered ways to ensure a minimum production of bioproducts such as acetaldehyde, citrate, glutarate, glycerol, phenol, and propanoate when growing the *N. aromaticivorans* strain using lignin-derived compounds as unique substrates.

## 1 Introduction

The increasing global demand for fuels and chemicals from non-renewable oil sources poses a severe problem as the world population continues to grow [1]. Lignocellulosic plant biomass, composed of cellulose, hemicellulose, and lignin, is our planet’s most abundant organic matter. It can potentially support a sustainable economy based on renewable resources [2]. However, unlike petroleum, lignocellulose is a highly oxygenated, solid, and heterogeneous substance [3].

Lignin is an amorphous heteropolymer containing mainly phenolic structures that differ in the number of methoxy groups attached to the aromatic rings: *syringyl* (S; two methoxy groups), *guaiacyl* (G; one methoxy group) and *p-hydroxyphenyl* (H; no methoxy groups) [4].

The pulp and paper industry produces around 50 million metric tons of lignin each year, and second-generation biorefineries generate approximately 1.5 kg of lignin per liter of cellulosic ethanol produced [5]. The utilization of lignin is currently limited due to its heterogeneous nature, and it is mainly used as a low-energy fuel [6]. This challenges the economic viability and sustainability of lignocellulosic biorefineries, including bioethanol plants, if lignin is mainly limited to low-value energy uses. Innovative approaches are required to transform lignin into high-value-added products, thus making it a recognized source of valuable materials [7].

Chemical techniques are used to break down plant biomass to obtain mixtures with a high proportion of low molecular weight phenolic compounds. This approach has attracted interest in valorizing lignin. Genetically modified microorganisms can convert these mixtures into a unique and valuable product [8].

Desirable characteristics of a microbial strain for developing lignin valorization strategies include its ability to metabolize most biomass-derived phenolic compounds and direct them to native convergent metabolic pathways [9]. Numerous microorganisms have been evaluated for these characteristics. Examples include genetically modified strains of *Corynebacterium glutamicum* [2], *Pseudomonas putida* KT2440 [9], [10], [11] and *Novosphingobium aromaticivorans* [12], 13].

This paper focuses on the bacterium *N. aromaticivorans* DSM12444 as a chassis microorganism for lignin valorization. This Gram-negative bacterium can efficiently utilize a wide variety of S-, G-, and H-phenols simultaneously. In addition, along with other sphingomonads, it possesses enzymes capable of breaking different bonds in the lignin polymer [14], [15], [16].

Our study is based on a genome-scale metabolic model (GEM) called iNovo479 [17], which is an *in silico* reconstruction of the complete metabolism of this microorganism. Along with the iNovo479 GEM model, they also introduced the production of certain products using *N. aromaticivorans* manufactured from vanillic acid (G-type phenol) as the only carbon source.

This study aims to find metabolic engineering strategies that can couple growth to synthesizing PDC (*2-pyrone-4-6-dicarboxylic acid*) and other chemicals in *N. aromaticivorans*. To achieve this, we use an algorithm based on genetic minimal cut sets (gMCS) [18], which helps us obtain all possible genetic interventions by knocking out at most four genes. With many interventions to consider, each strategy will be characterized and scored based on six criteria [19]. This will help us select the best candidates for successful metabolic engineering.

Furthermore, in our research paper, we list all the feasible genetic interventions that can be employed to produce valuable bioproducts such as acetaldehyde, citrate, glutarate, glycerol, phenol, and propanoate. We formulate a new algorithm that can identify genes involved in the biosynthesis of these compounds, potentially enhancing their accumulation.

The paper presents the following main findings: Firstly, we discover new genetic interventions that lead to the production of PDC using the iNovo479 model for the microbe *N. aromaticivorans*. Secondly, we identify several genetic interventions that can induce the production of acetaldehyde, citrate, glutarate, glycerol, phenol, and propanoate by using modified strains [17].

## 2 Materials and methods

### 2.1 Contraint-based modeling

A genome-scale metabolic model, also known as GEM, is a mathematical way of representing a biological system. GEM comprises three main components: the set of reactions denoted as *R*, the set of metabolites labeled as *M*, and the stoichiometric matrix named *S*. A vector *v* ∈ **R** ^*n*^ (where *n* is the number of reactions) can represent any possible network state.

Each vector *v*[*i*] component indicates the flux amount through reaction *r*_*i*_. The internal metabolites are assumed to be steady, which leads to the metabolite equilibrium equation.

(1) S⋅v=0

Two values, *lb*_*i*_ and *ub*_*i*_, are assigned to each reaction *r*_*i*_ to restrict its possible flux.

(2) $$lb_{i}\leq r_{i}\leq ub_{i}\;,\forall r_{i}\in R$$

A mode or state of the network is defined as a flux vector that satisfies equations (1) and (2). Two network states are considered equivalent if one is a non-negative multiple of the other. It is worth noting that these equivalent states will always be identified as the same mode, albeit with a slight abuse of notation. There are always infinite nonequivalent modes in non-trivial networks. The set of all possible network nodes is denoted as *C*, which forms the flux cone of the metabolic network.

(3) $$C=\left\{v\in R_{n}\;|\;S\cdot v=0,\;lb_{i}\leq r_{i}\leq ub_{i}\;,\forall r_{i}\in R\right\}$$

For any given mode *v* ∈ *C*, its support, supp(*v*), is defined as the reactions that appear with non-zero flux in *v*.

(4) $$\text{supp}\left(v\right)=\left\{r_{i}\in R\;|\;v_{i}\neq 0\right\}$$

Most models include a set of genes denoted by *G*. Each reaction *r* has an associated boolean expression called its gene-protein rule (GPR). The GPR specifies which genes must be activated for the flux through the reaction to be non-zero.

### 2.2 Coupled growth

The primary objective of metabolic network modeling is to comprehend and regulate the different behaviors exhibited by the network, with the ultimate goal of optimizing the production of bioproducts.

Combining metabolic engineering strategies can maximize flux through bioproduct synthesis. One practical approach is blocking pathways that compete with critical precursor synthesis or consume the bioproducts. A crucial step is identifying interventions that link growth (simulated by a lumped biomass reaction) with producing a specific bioproduct. This means that the synthesis of the product should occur regardless of the growth rate (strong coupling). To achieve this, sets of genes whose deactivation minimizes metabolic flux towards unwanted side products are identified. This enables the production of bioproducts even when cell growth is suboptimal or negligible [20].

The minimal cut set (MCS) method is a technique for identifying possible metabolic intervention strategies by targeting specific reactions. An MCS is the smallest set of reactions that can be inhibited (knocked out) to block certain undesired (target) flux distributions. In our specific problem, we are looking for MCSs that can block all flux vectors without bioproduct production coupled with growth. In simpler terms, we want to prevent any scenario where growth occurs without producing bioproducts.

The original MCS algorithm may not provide workable strategies at the gene level due to the one-to-many nature of GPR rules. Researchers have expanded MCS methods to address this limitation to obtain minimal gene deletion interventions, known as genetic minimal cut sets (gMCS). For further details, refer to [21], 22].

Identifying gMCSs is challenging and resource-intensive, especially for large networks requiring more than two or three gene knockouts. We have used a technique developed by the GACOP group to identify gMCSs [18]. After determining the gMCSs, we create a model by deactivating the genes within these sets. Using FBA techniques, we then analyze the solution space of these mutant strains to understand the effects of these knockouts better.

A second strategy to increase flux towards biosynthesis involves overexpressing rate-limiting enzymes, which produce intermediates specifically for biosynthesis, as well as regulatory enzymes and aromatic transporter proteins. However, genome-scale metabolic models (GEMs) do not permit the overexpression of genes, making it challenging to identify genes whose overexpression could enhance the production of bioproducts. A potential solution to this issue can be found in the Supplementary Material.

### 2.3 Ranking genetic strategies

Screening and evaluating multiple strategies is essential to determine the best candidates. Schneider and Klamt [19] proposed several criteria for characterizing growth-related intervention strategies. Here, we have adopted some of these criteria for the classification and scoring of the genetic strategies found. These are:

1. **Number of interventions required (int):** The experimental effort can be reduced by minimizing the required interventions.
2. **Biomass yield** *Y*_*x*/*s*_ defined as the ratio of the maximum growth rate to the sum of the substrate fluxes.
3. **Minimum product yield at maximum growth rate (min** ***Y***_***p*/*s***_ **/max x):** The point of maximum mutant performance after adaptive evolution. It involves maximizing the growth rate while minimizing the production of bioproducts at a given maximum growth rate.
4. **Minimum product yield (min** ***Y***_***p*/*s***_ **):** The product yield in the mutant when growth is sub-optimal.
5. **Requirement for anaerobic conditions (%O2):** To make scaling up growth easier, the mutant strain should consume less oxygen than the wild-type strain.
6. **Number of accessible metabolites:** When a disturbance in the middle or end of a pathway can cause a buildup of unwanted intermediate metabolites. To prevent this, it is better to interrupt the pathways at their branching points, limiting the overall number of metabolites produced.

Each intervention has been given a score based on specific criteria [19], as explained in the Supplementary Material.

### 2.4 iNovo479 model of *N. aromaticivorans*

All data and the GEM used for metabolic analysis of *N. aromaticivorans* were implemented in [17].

**Figure 1:**
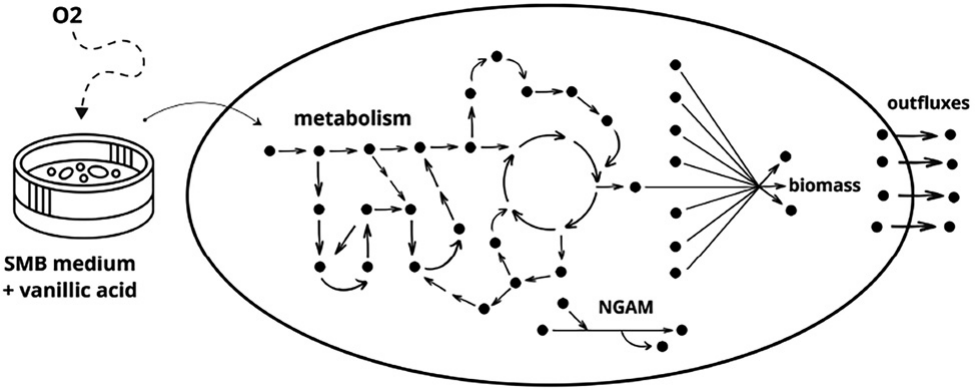
Model scheme.

iNovo479 was constructed using KEGG annotations predicted for the *N. aromaticivorans* DSM 12444 genome and annotation data available in the Integrated Microbial Genomes database. The model consists of 479 genes, 645 reactions, and 604 metabolites. Aromatic metabolism reactions and missing reactions in vital metabolic pathways were added manually based on the knowledge available in the literature. The biomass reaction in iNovo479 has been modified from the aerobic biomass reaction present in iRsp1095, a GEM created for another *α*-proteobacterium called *Rhodobacter sphaeroides*. The modification was done by incorporating laboratory-determined biomass composition data. Moreover, a non-growth-associated ATP maintenance reaction (NGAM) has been added to this model (refer to Figure 1 for details).

Metabolic engineering is necessary for the accumulation of bioproducts since, in the wild-type strain, all the energy obtained from the carbon source is devoted to the growth and maintenance of the organism, not to the biosynthesis of these products. Linz et al. [17] applied a genome engineering strategy [13] for the accumulation of *2-pyrone-4-6-dicarboxylic* acid (PDC), a compound with remarkable functional properties that is replacing terephthalic acid in polyester manufacturing [23]. On the other hand, a modified iNovo479 model was generated by introducing reactions that produced potentially valuable chemicals from intermediates generated by the metabolism of aromatics, such as glutarate, zeaxanthin, citrate, or propanoate.

The results obtained in [17] have been reproduced using COBRA and dFBA. The results we obtained agree in most cases, although there are slight differences in the rate of PDC production when the ratio of aromatic:glucose is 1:1 and 9:1.

Combining metabolic engineering strategies maximizes bioproduct synthesis by blocking competing pathways and consuming critical precursors.

To find gMCS for growth-coupled PDC production, glucose and vanillic acid were used as substrates. According to [13], the microorganism cannot produce PDC by using vanillic acid as the sole carbon source in the deletion strain. A proportion of glucose must be supplied to allow the microorganism to grow and produce PDC simultaneously. Even if a source of glucose is provided, the model does not use vanillic acid by default when its pathway to central carbon metabolites is blocked. We suggest its entry through a restriction based on a three-to-two molar aromatic-to-glucose ratio excess. This is the ratio at which the highest production rate is obtained [17].

In this paper, we use a modified model [17] to obtain genetic engineering strategies leading to an accumulation of bioproducts of industrial interest, including glutarate, acetaldehyde, citrate, glycerol, phenol, and propionate.

## 3 Results and discussion

The analysis of the model was performed using the COBRApy package [24] working within Jupyter-Notebook [25] and Python3.6 as the kernel [22]. For solving the associated LP problems, we have used Cplex solver [26]. All the tasks were run through Gacop’s Cluster, a group of different computing nodes forms. The research group of high-performance computer architecture (GACOP) of the University of Murcia (Spain) provided cluster support.

### 3.1 New genetic interventions to accumulate PDC from vanillic acid and glucose

In iNovo479, Linz et al. [17] introduced a technique to block the *ligI* gene for PDC accumulation using vanillic acid and glucose as substrates.

Our objective was to investigate new genetic interventions that were not previously explored. We have discovered seven intervention strategies (gMCS) with at least one and a maximum of 4 interventions. Table 1 displays the biomass yield, product yields, and the change in oxygen consumption compared to the wild-type conditions for each knockout. The initial row represents the wild-type (WT) phenotype, while the remaining rows indicate the intervention gene(s) to be deleted.

**Table 1:** Knockouts (gMCS) found for PDC from vanillic acid and glucose in a 3:2 ratio of vanillic to glucose.

Name	Strain	*Y*_*x*/*s*_	*Y*_*p*/*x*_	Min *Y*_*p*/*s*_ max *x*	Max *Y*_*p*/*s*_ max *x*	Min *Y*_*p*/*s*_	%O2
WT	WT	121.190	0.000	0.000	0.000	0.000	
gMCS1	Δ*ligI*	58.264	0.010	0.600	0.600	0.043	−19.417
gMCS2	Δ*ligK*	71.056	0.008	0.522	0.600	0.043	−19.449
gMCS3	Δ*ligJ*	71.056	0.008	0.522	0.600	0.043	−19.449
gMCS4	Δ*pckA*	89.229	0.004	0.362	0.464	0.043	−14.817
gMCS5	Δ*sucC*Δ*sucD*Δ*gcvT*Δ*cysE*	75.693	0.006	0.448	0.573	0.272	−19.574
gMCS6	Δ*sucC*Δ*sucD*Δ*gcvPB*Δ*cysE*	75.909	0.006	0.420	0.579	0.271	−19.519
gMCS7	Δ*sucC*Δ*sucD*Δ*gcvPA*Δ*cysE*	75.909	0.006	0.420	0.579	0.271	−19.519

*Y*_*x*/*s*_ (biomass yield, mgDW/mmol): biomass produced relative to substrate consumed. *Y*_*p*/*x*_ (product yield, mmol/mgDW): product formed about biomass produced. Min *Y*_*p*/*s*_/max *x* (mmol/mmol): minimum product yield at maximum growth rate. Max *Y*_*p*/*s*_/max *x* (mmol/mmol): maximum product yield at maximum growth rate. Min *Y*_*p*/*s*_ (mmol/mmol): minimum product yield. %O2: oxygen ratio increasing (positive sign) or decreasing (negative sign) relative to wild-type. The name of the strains corresponds to the uppercase delta symbol followed by the gene’s name to be deleted.

Figure 1 in Supplementary Material shows the scores of all interventions found for each bioproduct according to the criteria extracted from [19].

### 3.2 Obtaining other bioproducts of interest by modified strains of *N. aromaticivorans* from vanillic acid

In the modified iNovo479 model, vanillic acid is the sole carbon source (engineering strategies do not interrupt the degradation pathway from vanillic acid to the TCA cycle). It uses a series of enzymatic reactions to synthesize bioproducts of chemical interest from intermediates that the metabolism of aromatics could generate. The model does not tend to accumulate bioproducts as the energy derived from the carbon source is directed towards supporting growth. Therefore, to ensure minimal production, knockouts must be performed based on calculated gMCS.

We have found new genetic interventions to enable the production of 6 out of the 13 bioproducts [17]: acetaldehyde, citrate, glutarate, glycerol, phenol, and propanoate. Table 2 summarizes the corresponding results. This table highlights the number of interventions (gene knockouts) for each product along with the highest *Y*_*p*/*s*_ at the maximum growth rate and lowest *Y*_*p*/*s*_ values obtained.

**Table 2:** Summary of the results obtained for each bioproduct analyzed. The parameters of the highest-scoring interventions are shown. Vanillic acid is used as the sole carbon source.

Bioproduct	*Y*_*x*/*s*_	*Y*_*p*/*x*_	Min *Y*_*p*/*s*_ max *x*	Max *Y*_*p*/*s*_ max *x*	Min *Y*_*p*/*s*_	%O2
Glutarate	88.420	0.003	0.183	0.373	0.134	−0.265
Citrate	89.638	0.005	0.457	0.402	0.297	−15.531
Propionate	99.931	0.005	0.751	0.751	0.606	−5.719
Acetaldehyde	58.087	0.017	0.918	1.867	0.964	−3.412
Glycerol	84.356	0.007	1.111	1.111	0.654	−8.056
Phenol	115.870	0.001	3.018	3.018	0.148	−1.115

The results for knockouts of genes for these bioproducts are available in the Supplementary Material.

### 3.3 Genetic interventions to accumulate PDC from vanillic acid and glucose

**Figure 2:**
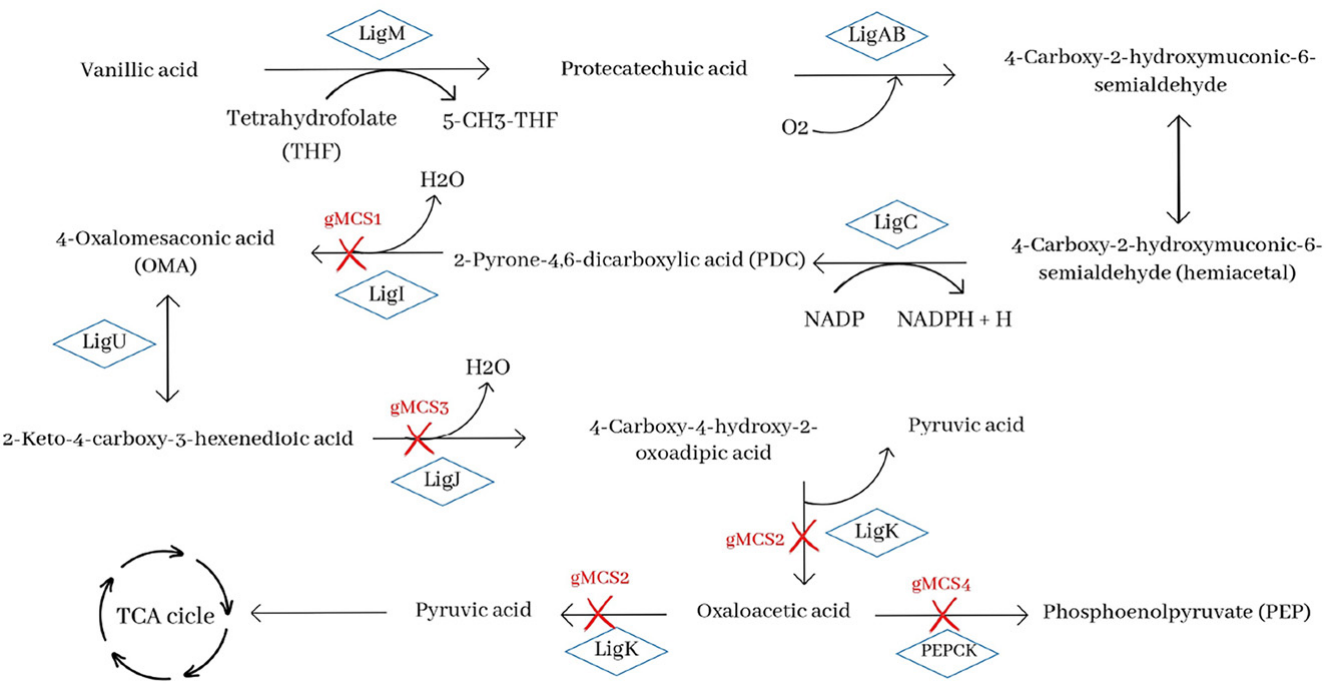
PDC synthesis pathway from vanillic acid in *Novosphingobium aromaticivorans*. Reactions blocked by gMCS 1, 2, 3, and 4 are marked with a red cross.

The pathway for PDC production is as follows: vanillic acid is converted to protocatechuate (PCA) by *tetrahydrofolate-dependent O-demethylase* (*LigM*) and catabolized via the PCA cleavage pathway. In this pathway, the aromatic ring of PCA is cleaved by PCA 4,5-dioxygenase (LigAB) to generate 4-carboxy-2-hydroxymuconate-6-semialdehyde (CHMS), which is then converted to 2-pyrone-4,6-dicarboxylate (PDC) by CHMS dehydrogenase (LigC). PDC is catabolized to oxaloacetate and pyruvate by reactions catalyzed by enzymes (LigI, LigU, LigJ, and LigK). The biosynthesis pathway is shown in Figure 2.

One of the genetic strategies obtained for PDC production is the inactivation of the *ligI* gene (gMSC1, Figure 2), which controls the reaction catalyzed by 2-pyrone-4,6-dicarboxylate hydrolase (LigI) and converts PDC to 4-oxalomesaconic acid (OMA). It also participates in the degradation of the latter compound. This strategy was also implemented [13], 17] for PDC accumulation using the same carbon source: vanillic acid and glucose.

Three other interventions involving the inactivation of a single gene have been obtained. Two of these genes are *ligK* and *ligJ* (gMSC2 and gMSC3, respectively), whose function is to control the reaction catalyzed by LigK (4-carboxy-4-hydroxy-2-oxoadipate aldolase) and an amidohydrolase (LigJ). Both are involved in the degradation of OMA to oxaloacetate and pyruvate. Although gMSC2 and gMSC3 have slightly higher scores than gMSC1, they have more accessible metabolites. LigK and LigJ disrupt the PDC-to-oxaloacetate pathway at an intermediate point, not at the branch point, as LigI does.

Blockage of *ligI* (gMSC1) *ligK* (gMSC2) or *ligJ* (gMSC3) targets the direct accumulation of PDC or its downstream metabolite, OMA, to decrease the flux of PDC catabolism into the tricarboxylic acid (TCA) cycle and increase its accumulation.

The fourth intervention is the inactivation of *pckA* gene. This gene encodes phosphoenolpyruvate carboxykinase (PEPCK), which catalyzes the oxaloacetate to phosphoenolpyruvate (PEP) reaction. This intervention results in a higher biomass yield as it does not directly interrupt the flow of PEP into the TCA cycle. However, it has a lower overall score as the product yield is lower. This intervention targets the accumulation of oxaloacetate and the decrease of PDC flux towards this compound.

Perez et al. [13] showed experimentally that disruption of *ligI* gene is sufficient to prevent PDC catabolism. Here, it has been proposed that, in addition to deletion of this gene, deletions of genes involved in the OMA to oxaloacetate pathway (*ligK* and *ligJ*) or decreased flux of PDC degradation (*pckA*) could also serve as a genetic strategy to maximize PDC production from vanillic acid.

As shown in Table 1, the strategy of blocking ligK or ligJ, as opposed to blocking ligI, produces a scenario in which a higher biomass yield (*Y*_*x*_/*s*) is observed, although with a slightly lower PDC yield (*Y*_*p*_/*x*). This could be because, by interrupting ligK- or ligJ-catalyzed reactions, the PDC degradation pathway is halted at an intermediate point, allowing the accumulation of downstream metabolites-primarily oxalomesaconic acid (OMA), which, instead of being wholly channeled into the TCA cycle, could be used to increase biomass yield (*Y*_*x*_/*s*) by incorporation into alternative metabolic pathways, thus compensating for the observed slight decrease in PDC yield (*Y*_*p*_/*x*).

In other words, although part of the substrate is “lost” in the form of OMA (and, potentially, 2-keto-4-carboxyl-hexanedioic acid), these intermediate metabolites could serve as a carbon source for growth. Furthermore, according to the ranking criteria used in the study [19], strategies based on ligK and ligJ inactivation score higher than ligI, as the balance between product yield and growth robustness is overall more favorable.

In summary, the preference for blocking ligK or ligJ lies in the fact that, although some of the substrate accumulates as downstream metabolites (which could be used as a carbon source for growth), the net effect is an increase in biomass, which favors the stability and efficiency of the PDC production process. It is important to emphasize that this hypothesis would require experimental validation to confirm whether, indeed, these metabolites can be redirected to provide energy for cell growth.

In addition, three interventions involving four gene knockouts have been found. These three interventions have three genes in common: *sucC*, *sucD*, and *cysE*.

Succinyl-CoA synthetase or succinate-CoA ligase is an enzyme that catalyzes the reversible reaction of succinate to succinyl-CoA in the TCA cycle. The *sucD* gene encodes the alpha subunit, while *sucC* encodes the beta subunit.

**Figure 3:**
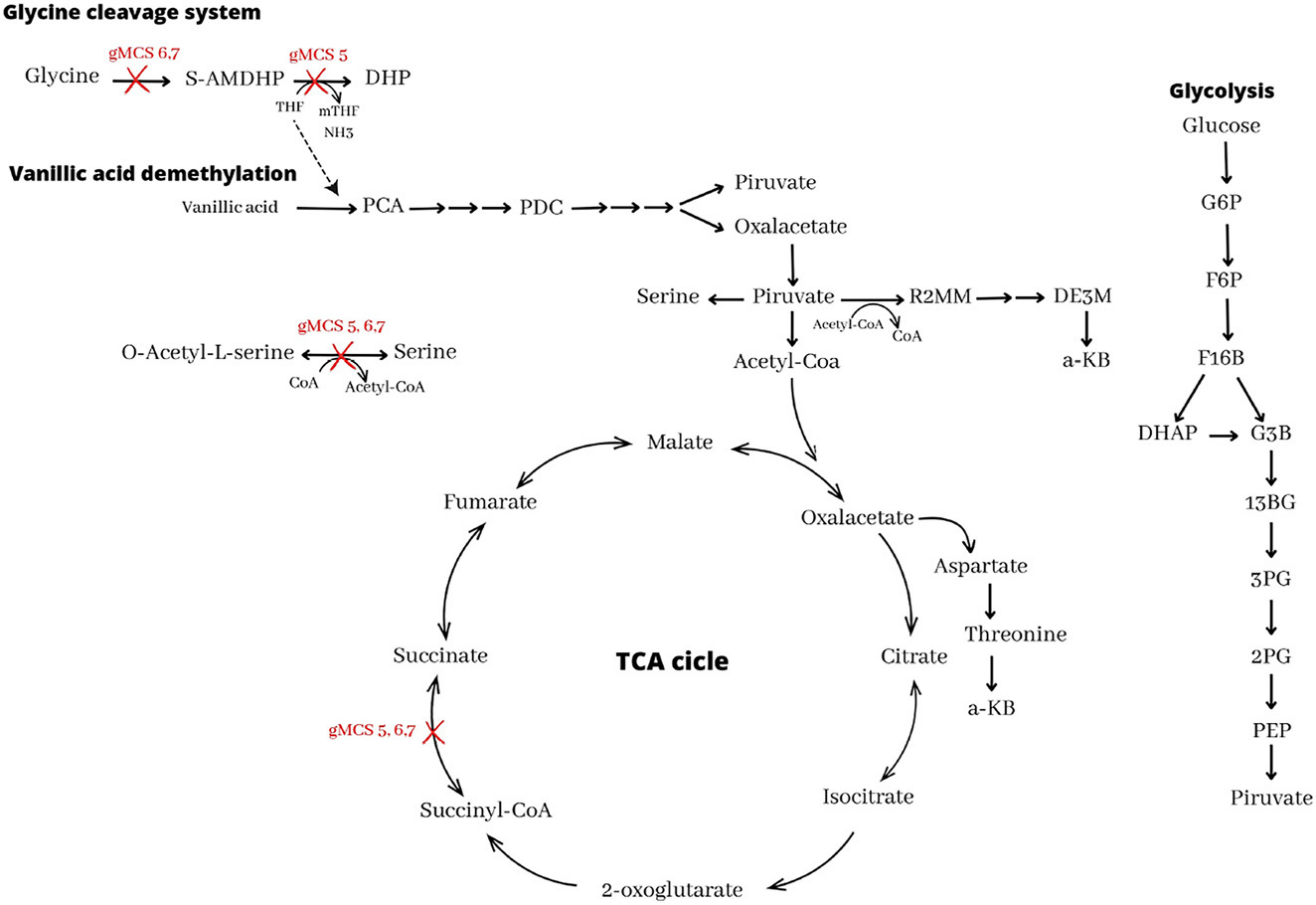
PDC synthesis pathway from vanillic acid in *Novosphingobium aromaticivorans*. Reactions blocked by gMCS 5,6, and 7 are marked with a red cross. S-AMDHP: S-aminomethyldihydrolipoylprotein; THF: tetrahydrofolate; mTHF: 5,10-methylenetetrahydrofolate; DHP: dihydrolipoylprotein; PCA: protocatechuic acid; DE3M: d-erythro-3-methylmalate; R2MM: R-2-methylmalate; a-KB: alpha-ketobutyrate; G6P: glucose-6-phosphate; F6P: fructose-6-phosphate; F16B: fructose-1,6-biphosphate; DHAP: dihydroxyacetone phosphate; G3P: glyceraldehyde 3-phosphate; 13BG; 1,3-biphosphoglycerate; 3PG: 3-phosphoglycerate; 2PG: 2-phosphoglycerate; PEP: phosphoenolpyruvate.

On the other hand, *cysE* encodes the enzyme serine O-acetyltransferase. This enzyme catalyzes the reversible reaction of serine with acetyl-CoA to give O-acetyl-l-serine. On the other hand, serine biosynthesis occurs via l-serine ammonium-lyase, with ammonium and pyruvate as substrates (Figure 3).

The other genes that appear (one in each genetic intervention) belong to the same biological process: the glycine cleavage system (GCS). This multi-enzyme system composed of four proteins (P, H, T, and L) catalyzes the reversible oxidation of glycine [27].

The *gcvT* gene (gMCS5) encodes the tetrahydrofolate aminomethyltransferase or T protein. It acts on GCS secondarily by degrading aminomethyl to ammonia in the presence of tetrahydrofolate (THF). On the other hand, the *gcvPB* and *gcvPA* genes (gMCS6 and gMCS7, respectively) encode lipoyl-protein oxidoreductase (P-protein), the first enzyme of the GCS system. With the deletion of succinyl-CoA synthetase, there is a decrease in the flux of the TCA cycle (Figure 3). This results in the accumulation of metabolites targeted to this cycle, including PDC and the metabolites included in its degradation pathway. The flux towards serine biosynthesis from pyruvate increases, as does glycolysis.

On the other hand, the formation of threonine from aspartate decreases, making synthesis rather than degradation to keto butyric acid the predominant fate of this essential amino acid protein. The route of its biosynthesis via pyruvate and acetyl-CoA is activated to recover the latter compound. The O-demethylation of vanillic acid by LigM uses THF as a cofactor (Figure 2), so the deletion of the gvcT gene and the consequent absence of the T protein will result in the accumulation of THF, thus increasing its flux towards vanillic acid demethylation. In addition, deletion of the P protein will block this pathway from the start and similarly lead to increased THF availability.

These three interventions result in higher biomass and product yields with lower oxygen consumption, outperforming single-gene interventions.

In gMCS5/6/7 strategies, the deletion of genes such as sucC, sucD, gcvT (or its variants gcvPB/gcvPA) and cysE disrupts the normal flow of the Krebs cycle, preventing metabolites downstream of PDC from being channeled to central metabolism. Thus, PDC accumulation would be favored, as the reactions that generally degrade or transform these metabolites are blocked.

In this scenario, it is essential to remember that vanillate is the primary source of PDC formation, whereas glucose is used exclusively for cell growth. Thus, even though glucose-derived pyruvate is not channeled into the Krebs cycle due to the blockage of this pathway, it can be employed through these alternative pathways to generate energy and, consequently, favor biomass synthesis.

Simulations predict that, in gMCS5/6/7, by preventing complete degradation of PDC, higher minimum PDC yields (min *Y*_*p*_/*s*) are obtained compared to other strategies. This parameter is defined as the minimum amount of product (in this case, PDC) obtained per mole of substrate consumed, measured under suboptimal growth conditions or at the point of maximum growth rate. This indicator is crucial because it ensures that, even in the least favorable scenarios for production, a minimum threshold of bioproduct synthesis is reached, allowing the robustness and feasibility of the metabolic engineering strategy to be assessed.

However, it is important to underline that this hypothesis is based on the simulations performed and the interpretation of the in silico models. It would be exciting for future studies to have experimental parts or simulations presenting metabolite profiles-for example, showing the fraction of vanillate converted to PDC in gMCS5/6/7 versus other strategies-to confirm whether, indeed, the accumulation of downstream metabolites translates into increased PDC formation and whether those metabolites can contribute to the generation of energy for growth.

### 3.4 Glutarate production

Glutaric acid is a metabolite of l-lysine catabolism in *Pseudomonas* species, in which l-lysine is converted to glutaric acid via the 5-amino valeric acid (VAA) pathway (Table 3). In order for *N. aromaticivorans* to produce this compound, four reactions involving four enzymes were introduced into iNovo479: a 2-oxidoreductase, a 5-aminopentanamide, an amidohydrolase, a 2-oxoglutarate aminotransferase, and an NAD+ oxidoreductase. From this modified GEM, genetic strategies for glutarate production were sought. As a result, three interventions with four genes per intervention were obtained. These three strategies are the same as those found for PDC production: deletion of the *sucC*, *sucD*, *cysE* and *gcvT*, *gcvPB* or *gcvPA* genes.

**Table 3:** Score for each of the genetic strategies for obtaining glutarate.

Name	Strain	Score
gMCS1	ΔsucCΔsucDΔgcvTΔcysE	2.0
gMCS2	ΔsucCΔsucDΔgcvPBΔcysE	2.0
gMCS3	ΔsucCΔsucDΔgcvPAΔcysE	2.0

**Figure 4:**
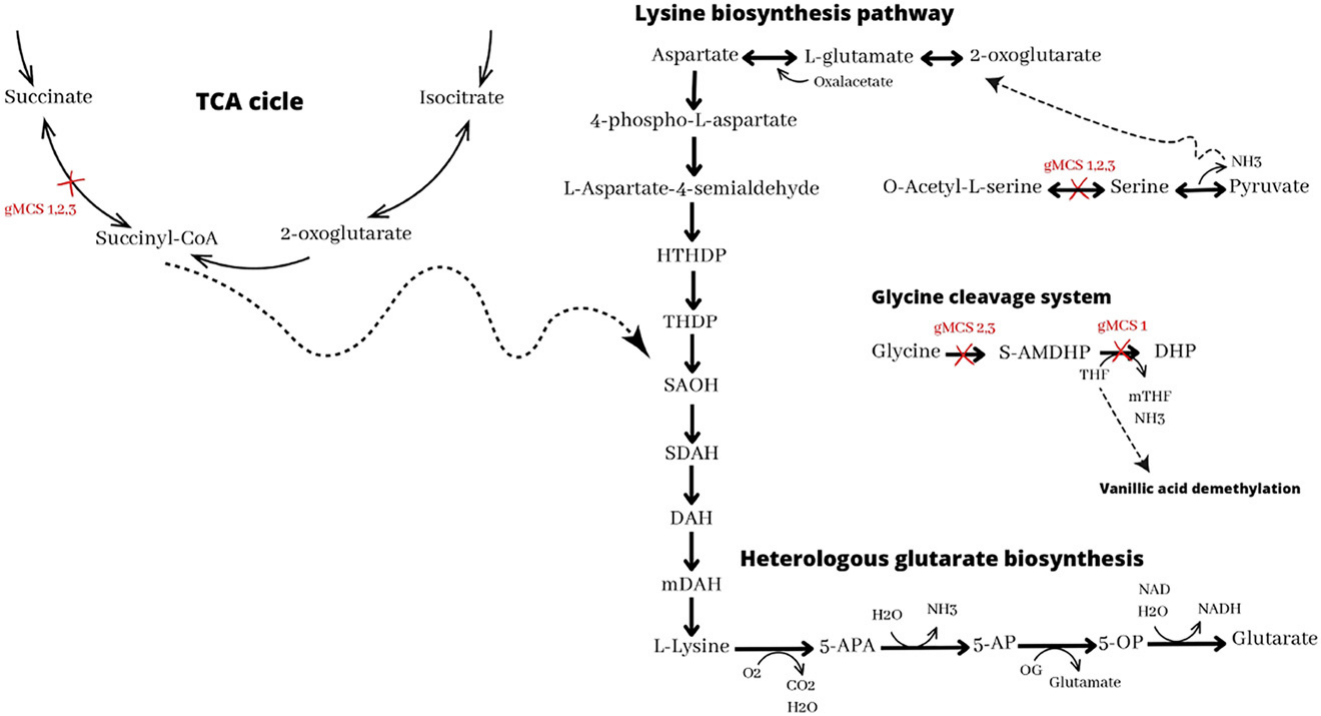
Glutarate synthesis pathway from vanillic acid in *Novosphingobium aromaticivorans*. Reactions blocked by gMCS 1,2 and 3 are marked with a red cross. HTHDP: (2S,4S)-4-hydroxy-2,3,4,5-tetrahydrodipicolinate; THDP: 2,3,4,5-tetrahydrodipicolinate; SAOH: N-succinyl-2-l-amino-6-oxoheptanedioate; SDAH: N-succinyl-LL-2,6-diaminoheptanedioate; DAH: LL-2,6-diaminoheptanedioate; mDAH: meso-2,6-diaminoheptanedioate; 5-APA: 5-aminopentanamide; 5-AP: 5-aminopentanoate; OG: 2-oxoglutarate; 5-OP: 5-oxopentanoate; S-AMDHP: S-aminomethyldihydrolipoylprotein; THF: tetrahydrofolate; mTHF: 5,10-methylenetetrahydrofolate; NH3: ammonia; DHP: dihydrolipoylprotein.

Succinyl-CoA is an essential compound in multiple metabolic pathways, including the TCA cycle and the lysine biosynthesis pathway shown in Figure 4. While it is possible for *Esterichia coli* to survive without succinyl-CoA, these cells have a more prolonged latency phase and slower growth rate, highlighting the importance of this compound in the TCA cycle [28].

Inactivation of these two genes could lead to a decrease in succinyl-CoA synthetase activity, accumulation of succinyl-CoA, and an increase in intracellular l-lysine available for glutaric acid production. Deletion of *cysE* would lead to an accumulation of serine, a decrease in its biosynthesis, and an increased bioavailability of ammonium and pyruvate. The ammonium influx would be directed to the synthesis of l-glutamate, a precursor of lysine. As in PDC production, the three genes that do not match in the interventions belong to the GCS biological process and aim to increase the bioavailability of THF.

On the other hand, all three strategies score equally (Figure 1 in Supplementary Material), although it is worth noting that strains with Δ*gcvPA* and Δ*gcvPB* with loss of function of Protein P have a lower number of accessible metabolites.

### 3.5 Citrate production

We have been able to obtain three strategies for citrate that are identical to those used for glutarate. Citrate is formed in the first reaction of the TCA cycle from oxaloacetate, acetyl-CoA, and water by citrate synthase (Table 4). With the deletion of serine O-acetyltransferase (*cysE*), acetyl-CoA accumulation would occur. In contrast, the deletion of succinyl-CoA synthetase (*sucC* and *sucD*) reduces the flux of the TCA cycle, and metabolites preceding succinyl-CoA in this cycle (including citrate) accumulate. On the other hand, deletion of the first two genes involved in the GCS will lead to THF accumulation. Citrate synthetase is inhibited by succinyl-CoA, which resembles acetyl-CoA and acts as a competitive inhibitor of acetyl-CoA and a non-competitive inhibitor of oxaloacetate, so blocking succinyl-CoA synthetase could reduce the yield of citrate production [29].

**Table 4:** Score for each of the genetic strategies for obtaining citrate.

Name	Strain	Score
gMCS1	ΔsucCΔsucDΔgcvTΔcysE	2.0
gMCS2	ΔsucCΔsucDΔgcvPBΔcysE	2.0
gMCS3	ΔsucCΔsucDΔgcvPAΔcysE	2.0

### 3.6 Propionate production

Propionic acid (propionate) is a commercially valuable carboxylic acid produced through microbial fermentation (Table 5). Propionic acid is mainly used in the food industry but has recently found applications in the cosmetics, plastics, and pharmaceutical industries. Propionate can be produced through several metabolic pathways, classified into three main groups: fermentative, biosynthetic, and amino acid catabolic pathways [30].

**Table 5:** The ten highest scoring genetic strategies for obtaining propionate.

Name	Strain	Score
gMCS2	Δ*pckA*	4.0
gMCS1	Δ*adk*	2.349
gMCS12	Δ*Saro_2568*Δ*pyk*Δ*Saro_0559*Δ*Saro_2259*	2.171
gMCS24	Δ*Saro_1894*Δ*pyk*Δ*Saro_RS09250*Δ*Saro_RS13605*	2.156
gMCS3	Δ*edd*Δ*pyk*Δ*Saro_RS09250*Δ*Saro_RS13605*	2.141
gMCS28	Δ*zwf*Δ*pyk*Δ*Saro_2679*Δ*Saro_0559*	2.095
gMCS27	Δ*zwf*Δ*pyk*Δ*Saro_RS09250*Δ*Saro_2259*	2.061
gMCS25	Δ*Saro_2568*Δ*pyk*Δ*Saro_2679*Δ*Saro_0559*	2.031
gMCS5	Δ*Saro_1894*Δ*pyk*Δ*Saro_RS09250*Δ*Saro_2259*	2.02
gMCS10	Δ*edd*Δ*pyk*Δ*Saro_0559*Δ*Saro_2259*	2.007

The metabolic pathway that allows propionate synthesis in *Novosphingobium arovativorans* is the degradation of threonine. This amino acid, synthesized from aspartate, is degraded to alpha-ketobutyrate and subsequently to propionate. In turn, aspartate can be obtained from oxaloacetate by 2-oxoglutarate aminotransferase.

**Figure 5:**
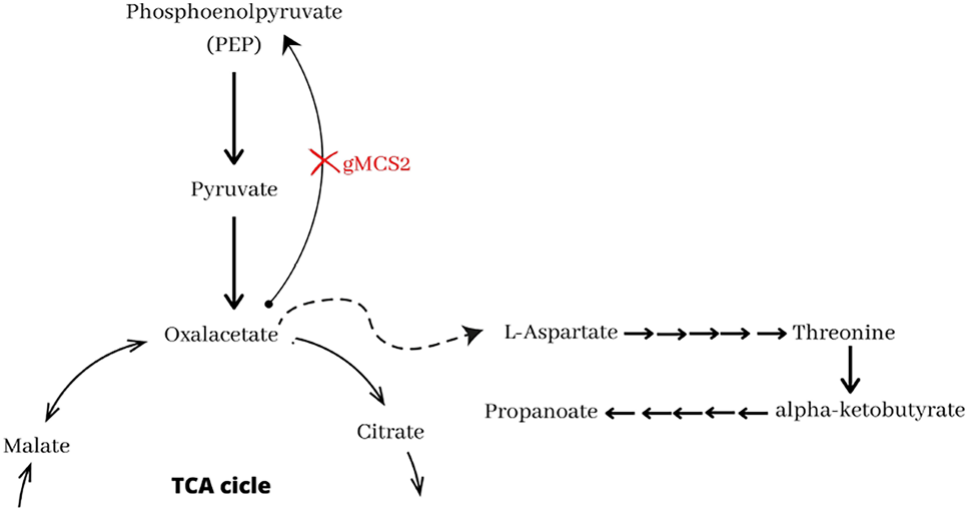
Propionate synthesis pathway from vanillic acid in *Novosphingobium aromaticivorans*. Reaction blocked by gMCS2 is marked with a red cross.

Twenty-nine interventions have been obtained, two of which are from a single gene and the rest from four genes (Figure 5).

The gMSC with the highest score is gMSC2 (Figure 1 in Supplementary Material), corresponding to deleting the *pckA* gene. Blocking this gene, which catalyzes the reaction of oxaloacetate to PEP by PEPCK, would lead to an accumulation of oxaloacetate and an increased flux toward aspartate synthesis. This intervention results in a higher biomass yield and a considerable increase in product yield compared to other gMSCs, although oxygen consumption is somewhat higher than under wild-type conditions. In *C. glutamicum* this gene was blocked for oxaloacetate and aspartate accumulation [31].

The other gMSC involving a single gene is gMSC1 (Δ*adk*) which encodes adenylate kinase (AK). The kinases represent a ubiquitous family of catalysts that play key roles in cellular metabolism, including the synthesis of RNA and DNA molecules and in energy metabolism. AK catalyzes the reversible transfer of the phosphate group between ATP and AMP, so its blockage leads to increased production of AMP, a metabolite required in the propionate synthesis reaction. AK is known to be essential for cell growth and survival [32], so blocking this gene may compromise the survival of *N. aromaticivorans*.

### 3.7 Acetaldehyde production

Acetaldehyde, like propionate, can be obtained through the metabolism of the amino acid threonine but with the action of different enzymes (Table 6). In this case, the action of treoninaldolase, an enzyme capable of splitting this amino acid into glycine and acetaldehyde, is involved. Threonine, in turn, is synthesized from aspartate.

**Table 6:** The ten highest scoring genetic strategies for obtaining acetaldehyde.

Name	Strain	Score
gMCS2	Δ*pckA*	3.797
gMCS1	Δ*purU*	3.685
gMCS27	Δ*zwf*Δ*pyk*Δ*Saro_2679*Δ*Saro_RS09250*	2.621
gMCS26	Δ*Saro_2568*Δ*pyk*Δ*Saro_2679*Δ*Saro_0559*	2.516
gMCS9	Δ*Saro_1894*Δ*pyk*Δ*Saro_RS09250*Δ*Saro_2259*	2.424
gMCS15	Δ*Saro_2568*Δ*pyk*Δ*Saro_0559*Δ*Saro_2259*	2.36
gMCS7	Δ*edd*Δ*pyk*Δ*Saro_RS09250*Δ*Saro_RS13605*	2.346
gMCS30	Δ*Saro_1894*Δ*pyk*Δ*Saro_2679*Δ*Saro_0559*	2.312
gMCS18	Δ*Saro_2568*Δ*pyk*Δ*Saro_RS09250*Δ*Saro_2259*	2.306
gMCS25	Δ*Saro_1894*Δ*pyk*Δ*Saro_RS09250*Δ*Saro_RS13605*	2.302

The highest scoring gMCS is gMCS2 (*pckA* (Figure 1 in Supplementary Material), which encodes PEPCK. Its deletion will lead to an accumulation of oxaloacetate, an increase in aspartate synthesis by the action of 2-oxoglutarate aminotransferase, and a consequent increase in flux to threonine synthesis.

Another proposed strategy is to block the glycine formation reaction (gMSC3, *glyA*). This increases the flux towards its synthesis from threonine metabolism, obtaining acetaldehyde from its degradation.

### 3.8 Glycerol production

Twenty-four strategies for maximizing glycerol synthesis are obtained, all formed by cutsets of 4 genes. The highest scoring is gMCS11 (Figure 1 in Supplementary Material), with the highest minimum product yield at maximum biomass (Table 7).

**Table 7:** The ten highest scoring genetic strategies for obtaining glycerol.

Name	Strain	Score
gMCS11	Δ*pyk*Δ*zwf*Δ*Saro_0559*Δ*Saro_2259*	3.053
gMCS5	Δ*zwf*Δ*pyk*Δ*Saro_RS09250*Δ*Saro_RS13605*	2.755
gMCS19	Δ*Saro_1894*Δ*pyk*Δ*Saro_RS09250*Δ*Saro_RS13605*	2.75
gMCS18	Δ*Saro_2568*Δ*pyk*Δ*Saro_0559*Δ*Saro_RS13605*	2.023
gMCS10	Δ*edd*Δ*pyk*Δ*Saro_0559*Δ*Saro_RS13605*	1.891
gMCS9	Δ*Saro_2568*Δ*pyk*Δ*Saro_0559*Δ*Saro_2259*	1.82
gMCS2	Δ*edd*Δ*pyk*Δ*Saro_2679*Δ*Saro_RS09250*	1.767
gMCS17	Δ*Saro_1894*Δ*pyk*Δ*Saro_0559*Δ*Saro_2259*	1.734
gMCS23	Δ*zwf*Δ*pyk*Δ*Saro_2679*Δ*Saro_0559*	1.722
gMCS16	Δ*pyk*Δ*zwf*Δ*Saro_0559*Δ*Saro_RS13605*	1.718

The gene repeated in all interventions is *pyk*. This gene encodes pyruvate kinase, which catalyzes the reaction of pyruvate formation from PEP. Glycerol is formed from 2-phospho-d-glycerate, and the action of 2-phospho-d-glycerate hydro-lyase with PEP as substrate synthesizes this metabolite. Therefore, *pyk* blockage targets the accumulation of PEP and an increase in glycerol synthesis. The only amplification target genes lead to the synthesis of sn-glycerol-3-phosphate, the glycerol precursor (Table 12 in Supplementary Material).

### 3.9 Phenol production

Twenty-four strategies consisting of cutsets of 4 genes are obtained. The highest scoring is gMCS18 (Figure 1 in Supplementary Material), with the highest minimum product yield at maximum biomass. As in glycerol production, the gene repeated in all interventions (maximum overlap) is *pyk*, which encodes pyruvate kinase. Phenol is formed from tyrosine from the reaction catalyzed by the heterologous enzyme l-tyrosine phenol-lyase. Tyrosine is synthesized through a series of reactions starting with the reaction of PEP with Shikimate 3-phosphate, catalyzed by 3-phosphoshikimate 5-O-(1-carboxyvinyl)-transferase. The blockage of *pyk* and the resulting accumulation of PEP will increase this compound’s flux towards tyrosine and phenol formation (Table 8).

**Table 8:** The ten highest scoring genetic strategies for obtaining phenol.

Name	Strain	Score
gMCS18	Δ*edd*Δ*pyk*Δ*Saro_0559*Δ*Saro_RS13605*	2.208
gMCS6	Δ*Saro_1894*Δ*pyk*Δ*Saro_0559*Δ*Saro_RS13605*	2.207
gMCS13	Δ*Saro_1894*Δ*pyk*Δ*Saro_0559*Δ*Saro_2259*	2.205
gMCS1	Δ*edd*Δ*pyk*Δ*Saro_2679*Δ*Saro_0559*	2.186
gMCS24	Δ*pyk*Δ*zwf*Δ*Saro_0559*Δ*Saro_RS13605*	2.148
gMCS5	Δ*pyk*Δ*zwf*Δ*Saro_2679*Δ*Saro_0559*	1.974
gMCS15	Δ*zwf*Δ*pyk*Δ*Saro_RS09250*Δ*Saro_RS13605*	1.526
gMCS7	Δ*Saro_1894*Δ*pyk*Δ*Saro_RS09250*Δ*Saro_2679*	1.49
gMCS12	Δ*Saro_2568*Δ*pyk*Δ*Saro_0559*Δ*Saro_2259*	1.46
gMCS16	Δ*edd*Δ*pyk*Δ*Saro_RS09250*Δ*Saro_2679*	1.375

**Figure 6:**
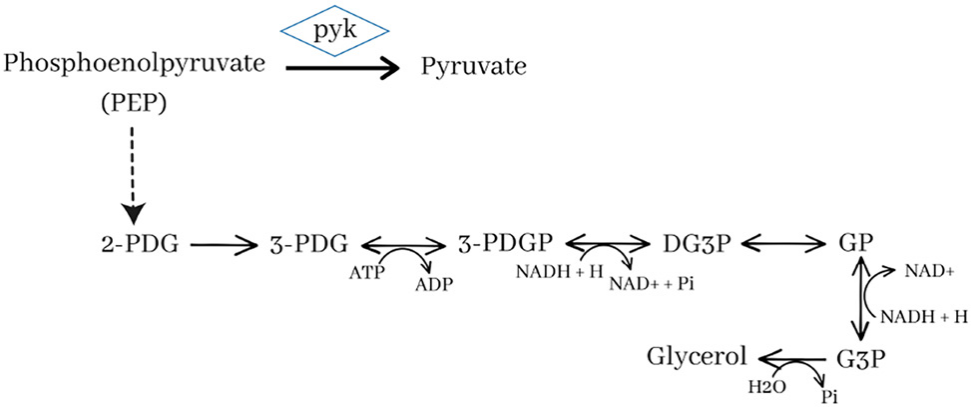
Glycerol synthesis pathway from vanillic acid in *Novosphingobium aromaticivorans*. 2-PDG: 2-phospho-d-glycerate; 3-PDG: 3-phospho-d-glycerate; 3-PDGP: 3-phospho-d-glycerol phosphate; DG3P: d-glyceraldehyde 3-phosphate; GP: glycerone phosphate; G3P: sn-glycerol 3-phosphate; Pi: orthophosphate.

In all bioproduction strategies, there is an accumulation of formate, a metabolite resulting from the demethylation of vanillic acid. This format forms reducing power (NADH) through the reaction catalyzed by an NAD+ oxidoreductase, thus compensating for reducing carbon flux by the TCA cycle. In addition, decreasing power is required to form some bioproducts such as glycerol (Figure 6).

### 3.10 General discussion

In this project, vanillic acid has been used as a carbon source for synthesizing bioproducts in the same way as implemented in [17]. However, there is a wide range of aromatic compounds that can be obtained from lignin depolymerization besides vanillic acid: *p*-hydroxybenzoic acid, syringic acid, syringaldehyde, protocatechuic acid, vanillin, ferulic acid, etc. For microbial valorization, the depolymerization of lignin into these low molecular weight compounds is crucial to ensure their bioavailability for further valorization by *N. aromaticivorans*. On the other hand, it is also of utmost importance to tailor depolymerization strategies to produce a suitable product spectrum. For *N. aromaticivorans* this spectrum is quite broad: it has been experimentally demonstrated to degrade up to 17 different aromatic compounds [33].

**Figure 7:**
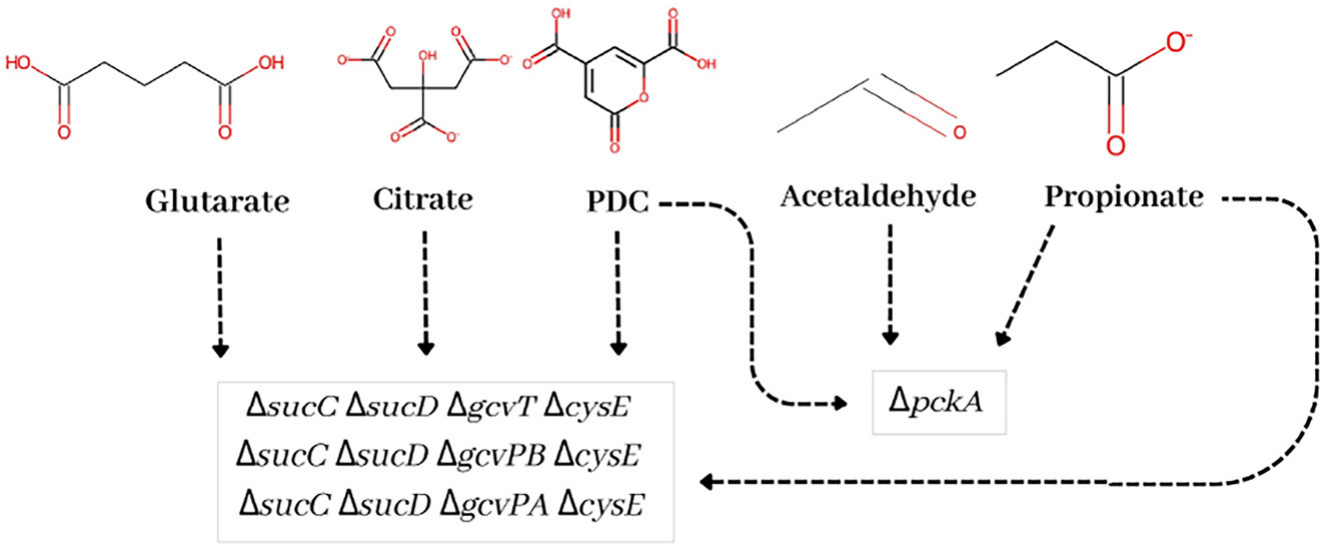
Genetic strategies that are common in different bioproducts.

We found 120 intervention strategies (gMCS) ranging from a minimum of 1 to a maximum of 4 genes that guarantee a minimum production of bioproducts while growing the *N. aromaticivorans* strain. Three of these genetic interventions are repeated to produce four different bioproducts. These strategies correspond to the blocking of genes encoding serine O-acetyltransferase (*cysE*), succinyl-CoA synthetase (*sucC* and *sucD*), and the P-protein (*gcvPA* and *gcvPB*) or T-protein (*gcvT*) of GCS (see Figure 7), which are common in the production of PDC, glutarate, citrate and propionate. These strategies target the accumulation of TCA cycle intermediates, a decrease in TCA cycle flux, and an increase in THF bioavailability. The other strategy is blocking the *pckA* gene that encodes phosphoenolpyruvate carboxykinase, which is involved in producing PDC, propionate, and acetaldehyde. This strategy seeks to accumulate oxaloacetate, either to decrease the flux from PDC to oxaloacetate (in the case of PDC) or to increase the flux to threonine synthase, the precursor of acetaldehyde and propionate.

## 4 Conclusions

Lignin is the second most abundant plant biopolymer on Earth, and massive amounts are generated in industries as waste material. Bacterial metabolic networks that mediate the degradation of various aromatic compounds are considered excellent candidates for integrating lignin into a biotechnological value chain.

Genome-scale metabolic models (GEM) can be used to search for genetic interventions that can produce desired compounds from lignin. This approach has several significant advantages over performing these genetic interventions one by one in the laboratory: it considerably reduces costs, as only the GEM needs to be constructed *in silico*, and it helps to accelerate the development of efficient strains for bio-based production processes as it allows us to visualize the phenotype of each mutated strain rapidly.

The GEM used in this work for metabolic engineering is iNovo479, which simulates the metabolism of *N. aromaticivorans* DSM12444. This microorganism can use a lignin-derived compound, vanillic acid, for the growth-coupled production of 2-pyrone-4,6-dicarboxylic acid (PDC) and other compounds of chemical interest. These strategies are obtained by combining genetic minimal cut sets (gMCS), i.e., sets of genes whose simultaneous inhibition forces the model to generate the desired by-products.

We have discovered seven new interventions involving no more than four genes in PDC production. Although these new strategies have a lower PDC yield on a biomass-produced basis (*Y*_*p*/*x*_) compared to the previously suggested *ligI* gene blocking [13], higher yields (*Y*_*x*/*s*_) are obtained with these strategies. Furthermore, the minimum PDC yield obtained from the strategy involving the deletion of four genes (gMCS 5, 6, and 7) is considerably higher (min *Y*_*p*/*s*_). This guarantees excellent PDC yield even under suboptimal conditions.

To select the best strategies, we followed a previously proposed criteria [19] and scored each method according to these criteria. The highest scoring strategies involve the deletion of *ligK* and *ligJ*, strategies not considered in previous literature.

Another main contribution of this work is the production of high-value compounds from lignin. Numerous studies have used different mutated microorganisms to produce these bioproducts from large amounts of glucose [34]. Consumption of this carbon source from the starch hydrolysis reaction creates competition for food with humans. Therefore, it is essential to exploit alternative renewable carbon sources, such as lignin, thus improving productivity in the synthesis of bioproducts and making the fermentation process more cost-effective [35].

Finally, the paper presents a new algorithm for detecting genes involved in the biosynthesis of products that could increase expression to accumulate compounds of interest.

In future work, we plan to develop an experimental phase necessary to verify that the *in silico* results correspond to reality. Thanks to the GEM models and the algorithm developed in this paper, we expect to confirm *in vitro* the finding of genetic interventions to ensure the production of high-value-added compounds such as PDC, acetaldehyde, citrate, glutarate, glycerol, phenol, and propanoate.

## Supplementary Material

Supplementary Material Details
