# Supplementary material for: Obtaining PDC and other high-added value products from lignin by in silico genetic engineering in Novosphingobium aromaticivorans
Source: J Integr Bioinform. 2026 Feb 25;22(3):20240059. doi: 10.1515/jib-2024-0059 (PMC13066346; doi:10.1515/jib-2024-0059)
Supplement: Supplementary file 1 — Supplementary Material Details [file j_jib-2024-0059_suppl_001.pdf]

1 Knockout strategies for the production of different bioproducts

Table 1 Knockouts for glutarate from vanillic acid. $Y_{x/s}$ (biomass yield, mgDW/mmol): biomass produced relative to substrate consumed. $Y_{p/x}$ (product yield, mmol/mgDW): product formed in relation to biomass produced. Min $Y_{p/s}$/max x (mmol/mmol): minimum product yield at maximum growth rate. Max $Y_{p/s}$/max x (mmol/mmol): maximum product yield at maximum growth rate. Min $Y_{p/s}$ (mmol/mmol): minimum product yield. %O2: oxygen ratio increasing (positive sign) or decreasing (negative sign) relative to wild-type. The name of the strains corresponds to the uppercase delta symbol followed by the name of the gene to be deleted.

Name	Strain	$Y_{x/s}$	$Y_{p/x}$	min $Y_{p/s}$ max x	max $Y_{p/s}$ max x	min $Y_{p/s}$	%O2
WT	WT	123.797665	0.000000	0.000000	0.000000	0.000000	
gMCS1	ΔsucCΔsucDΔgcvTΔcysE	91.034245	0.003131	0.181155	0.364657	0.140374	0.007900
gMCS2	ΔsucCΔsucDΔgcvPBΔcysE	88.419923	0.003193	0.182889	0.373063	0.133772	-0.264626
gMCS3	ΔsucCΔsucDΔgcvPAΔcysE	88.419923	0.003193	0.182889	0.373063	0.133772	-0.264626

Table 2 Knockouts for citrate from vanillic acid. $Y_{x/s}$ (biomass yield, mgDW/mmol): biomass produced relative to substrate consumed. $Y_{p/x}$ (product yield, mmol/mgDW): product formed in relation to biomass produced. Min $Y_{p/s}$/max x (mmol/mmol): minimum product yield at maximum growth rate. Max $Y_{p/s}$/max x (mmol/mmol): maximum product yield at maximum growth rate. Min $Y_{p/s}$ (mmol/mmol): minimum product yield. %O2: oxygen ratio increasing (positive sign) or decreasing (negative sign) relative to wild-type. The name of the strains corresponds to the uppercase delta symbol followed by the name of the gene to be deleted.

Name	Strain	$Y_{x/s}$	$Y_{p/x}$	min $Y_{p/s}$ max x	max $Y_{p/s}$ max x	min $Y_{p/s}$	%O2
WT	WT	129.105162	0.000000	0.000000	0.000000	0.000000	
gMCS1	ΔsucCΔsucDΔgcvTΔcysE	87.219005	0.005108	0.417737	0.5812998	0.296509	-17.137945
gMCS2	ΔsucCΔsucDΔgcvPBΔcysE	89.637501	0.005325	0.457038	0.4017063	0.296509	-15.530961
gMCS3	ΔsucCΔsucDΔgcvPAΔcysE	89.637501	0.005325	0.457038	0.4017063	0.296509	-15.530961

Table 3 Knockouts for propionate from vanillic acid. $Y_{x/s}$ (biomass yield, mgDW/mmol): biomass produced relative to substrate consumed. $Y_{p/x}$ (product yield, mmol/mgDW): product formed in relation to biomass produced. Min $Y_{p/s}$/max x (mmol/mmol): minimum product yield at maximum growth rate. Max $Y_{p/s}$/max x (mmol/mmol): maximum product yield at maximum growth rate. Min $Y_{p/s}$ (mmol/mmol):minimum product yield. %O2: oxygen ratio increasing (positive sign) or decreasing (negative sign) relative to wild-type. The name of the strains corresponds to the uppercase delta symbol followed by the name of the gene to be deleted.

Name	Strain	$Y_{x/s}$	$Y_{p/x}$	min $Y_{p/s}$ max x	max $Y_{p/s}$ max x	min $Y_{p/s}$	%O2
WT	WT	129.273473	0.000000	0.000000	0.000000	0.000000	
gMCS1	Δadk	101.276523	0.002267	0.229601	0.434989	0.229576	-7.247543
gMCS2	$\Delta pckA$	119.005456	0.005918	1.662753	1.662753	0.863589	2.976452
gMCS3	$\Delta edd\Delta pyk\Delta Saro\_RS09250\Delta Saro\_RS13605$	100.911770	0.004976	0.691427	0.691427	0.607964	-5.550477
gMCS4	$\Delta edd\Delta pyk\Delta Saro\_2679\Delta Saro\_RS09250$	75.482776	0.007705	0.564751	0.803675	0.602840	-11.322668
gMCS5	$\Delta Saro\_1894\Delta pyk\Delta Saro\_RS09250\Delta Saro\_2259$	76.602800	0.008522	0.617214	0.634462	0.652808	-10.721133
gMCS6	$\Delta Saro\_1894\Delta pyk\Delta Saro\_2679\Delta Saro\_RS09250$	72.107801	0.008311	0.430139	0.894359	0.604721	-10.786028
gMCS7	$\Delta sucC\Delta sucD\Delta gcvT\Delta cysE$	109.485413	0.003255	0.322221	0.398967	0.325934	-5.225149
gMCS8	$\Delta zwf\Delta pyk\Delta Saro\_RS09250\Delta Saro\_RS13605$	72.439042	0.008266	0.411042	0.743060	0.600534	-10.711785
gMCS9	$\Delta edd\Delta pyk\Delta Saro\_RS09250\Delta Saro\_2259$	80.736905	0.007258	0.255981	0.824580	0.586125	-9.224425
gMCS10	$\Delta edd\Delta pyk\Delta Saro\_0559\Delta Saro\_2259$	74.788120	0.008830	0.595814	0.835311	0.660459	-11.120415
gMCS11	$\Delta edd\Delta pyk\Delta Saro\_2679\Delta Saro\_0559$	73.914590	0.008077	0.596997	0.670908	0.597058	-10.450772
gMCS12	$\Delta Saro\_2568\Delta pyk\Delta Saro\_0559\Delta Saro\_2259$	99.931306	0.005202	0.750878	0.750878	0.605503	-5.719020
gMCS13	$\Delta sucC\Delta sucD\Delta gcvPA\Delta cysE$	111.360014	0.003246	0.351465	0.351465	0.330028	-6.334316
gMCS14	$\Delta edd\Delta pyk\Delta Saro\_0559\Delta Saro\_RS13605$	71.268860	0.008496	0.605478	0.808022	0.605478	-10.982009
gMCS15	$\Delta pyk\Delta zwf\Delta Saro\_0559\Delta Saro\_2259$	87.719111	0.006342	0.662541	0.424705	0.609347	-7.612266
gMCS16	$\Delta Saro\_2568\Delta pyk\Delta Saro\_RS09250\Delta Saro\_2259$	79.523365	0.007320	0.506143	0.855230	0.582089	-9.523639
gMCS17	$\Delta Saro\_2568\Delta pyk\Delta Saro\_2679\Delta Saro\_RS09250$	70.617844	0.008581	0.518817	0.760479	0.606055	-11.077090
gMCS18	$\Delta sucC\Delta sucD\Delta gcvPB\Delta cysE$	111.360014	0.003246	0.351465	0.351465	0.330028	-6.334316
gMCS19	$\Delta Saro\_1894\Delta pyk\Delta Saro\_0559\Delta Saro\_RS13605$	87.694176	0.006297	0.492295	1.010914	0.503354	-7.796319
gMCS20	$\Delta Saro\_2568\Delta pyk\Delta Saro\_RS09250\Delta Saro\_RS13605$	71.852210	0.008376	0.579916	0.734283	0.601869	-10.842190
gMCS21	$\Delta pyk\Delta zwf\Delta Saro\_0559\Delta Saro\_RS13605$	81.947032	0.007048	0.638685	0.560789	0.609778	-8.886697
gMCS22	$\Delta Saro\_1894\Delta pyk\Delta Saro\_0559\Delta Saro\_2259$	73.920228	0.008076	0.511491	0.837473	0.595448	-10.449653
gMCS23	$\Delta Saro\_2568\Delta pyk\Delta Saro\_0559\Delta Saro\_RS13605$	78.477976	0.007262	0.588201	0.833810	0.592487	-9.500136
gMCS24	$\Delta Saro\_1894\Delta pyk\Delta Saro\_RS09250\Delta Saro\_RS13605$	92.133142	0.006372	0.712641	0.712641	0.654319	-7.090176
gMCS25	$\Delta Saro\_2568\Delta pyk\Delta Saro\_2679\Delta Saro\_0559$	74.668797	0.008763	0.654297	0.836644	0.654394	-11.052649
gMCS26	$\Delta zwf\Delta pyk\Delta Saro\_2679\Delta Saro\_RS09250$	73.043820	0.008165	0.539243	0.572278	0.605535	-10.593679
gMCS27	$\Delta zwf\Delta pyk\Delta Saro\_RS09250\Delta Saro\_2259$	97.767088	0.005282	0.673074	0.673074	0.599037	-5.714224
gMCS28	$\Delta zwf\Delta pyk\Delta Saro\_2679\Delta Saro\_0559$	95.879271	0.005280	0.662352	0.662352	0.596784	-6.914364
gMCS29	$\Delta Saro\_1894\Delta pyk\Delta Saro\_2679\Delta Saro\_0559$	71.382322	0.008475	0.532481	0.839393	0.605446	-10.960815

Table 4 Knockouts for acetaldehyde from vanillic acid. $Y_{x/s}$ (biomass yield, mgDW/mmol): biomass produced relative to substrate consumed. $Y_{p/x}$ (product yield, mmol/mgDW): product formed in relation to biomass produced. Min $Y_{p/s}$/max x (mmol/mmol): minimum product yield at maximum growth rate. Max $Y_{p/s}$/max x (mmol/mmol): maximum product yield at maximum growth rate. Min $Y_{p/s}$ (mmol/mmol):minimum product yield. %O2: oxygen ratio increasing (positive sign) or decreasing (negative sign) relative to wild-type. The name of the strains corresponds to the uppercase delta symbol followed by the name of the gene to be deleted.

Name	Strain	$Y_{x/s}$	$Y_{p/x}$	min $Y_{p/s}$ max x	max $Y_{p/s}$ max x	min $Y_{p/s}$	%O2
WT	WT	116.793442	0.000000	0.000000	0.299565	0.000000	
gMCS1	Δ*purU*	58.087060	0.016617	0.918193	1.866614	0.963895	-3.411660
gMCS2	Δ*pckA*	73.686576	0.010947	0.755712	0.789095	0.789954	-7.285915
gMCS3	Δ*glyA*	108.010978	0.001024	0.110584	0.416327	0.110582	0.096274
gMCS4	Δ*Saro_1100*Δ*Saro_2259*	131.297433	0.001140	0.177210	0.128371	0.144815	5.020273
gMCS5	Δ*Saro_1100*Δ*Saro_RS13605*	125.005784	0.000919	0.174914	0.174914	0.146916	1.548465
gMCS6	Δ*Saro_1100*Δ*Saro_2679*	135.180001	0.001117	0.189195	0.189195	0.146957	4.637098
gMCS7	Δ*edd*Δ*pyk*Δ*Saro_RS09250*Δ*Saro_RS13605*	93.658776	0.006048	0.522674	1.103087	0.566405	-5.319667
gMCS8	Δ*edd*Δ*pyk*Δ*Saro_2679*Δ*Saro_RS09250*	114.611619	0.004350	0.552923	0.552923	0.509145	0.068291
gMCS9	Δ*Saro_1894*Δ*pyk*Δ*Saro_RS09250*Δ*Saro_2259*	94.026945	0.006095	0.574002	0.660098	0.572482	-5.345463
gMCS10	Δ*Saro_1894*Δ*pyk*Δ*Saro_2679*Δ*Saro_RS09250*	101.627001	0.004695	0.480552	0.671068	0.500901	-1.760741
gMCS11	Δ*zwf*Δ*pyk*Δ*Saro_RS09250*Δ*Saro_RS13605*	101.059796	0.005268	0.576964	0.576964	0.520090	-1.892817
gMCS12	Δ*edd*Δ*pyk*Δ*Saro_RS09250*Δ*Saro_2259*	89.015204	0.005781	0.362066	0.874982	0.515694	-4.637098
gMCS13	Δ*edd*Δ*pyk*Δ*Saro_0559*Δ*Saro_2259*	87.022763	0.005940	0.525949	0.864148	0.520075	-4.830494
gMCS14	Δ*edd*Δ*pyk*Δ*Saro_2679*Δ*Saro_0559*	104.212708	0.005015	0.532700	0.297171	0.505377	-2.083404
gMCS15	Δ*Saro_2568*Δ*pyk*Δ*Saro_0559*Δ*Saro_2259*	108.568082	0.004612	0.606638	0.606638	0.520131	-2.502665
gMCS16	Δ*edd*Δ*pyk*Δ*Saro_0559*Δ*Saro_RS13605*	89.014152	0.005782	0.480561	0.827543	0.505678	-4.637081
gMCS17	Δ*pyk*Δ*zwf*Δ*Saro_0559*Δ*Saro_2259*	97.136714	0.004811	0.561394	0.561394	0.520075	-2.438575
gMCS18	Δ*Saro_2568*Δ*pyk*Δ*Saro_RS09250*Δ*Saro_2259*	105.375003	0.005132	0.594176	0.594176	0.525078	-2.461809
gMCS19	Δ*Saro_2568*Δ*pyk*Δ*Saro_2679*Δ*Saro_RS09250*	89.705297	0.005682	0.557930	0.485102	0.520808	-4.461411
gMCS20	Δ*Saro_1894*Δ*pyk*Δ*Saro_0559*Δ*Saro_RS13605*	86.909806	0.005952	0.517300	1.249282	0.517357	-4.904918
gMCS21	Δ*Saro_2568*Δ*pyk*Δ*Saro_RS09250*Δ*Saro_RS13605*	87.426403	0.005902	0.523313	0.841390	0.518567	-4.835639
gMCS22	Δ*pyk*Δ*zwf*Δ*Saro_0559*Δ*Saro_RS13605*	102.923397	0.004906	0.568548	0.568548	0.514454	-1.975211
gMCS23	Δ*Saro_1894*Δ*pyk*Δ*Saro_0559*Δ*Saro_2259*	89.014319	0.005781	0.561759	1.153688	0.526108	-4.636780
gMCS24	Δ*Saro_2568*Δ*pyk*Δ*Saro_0559*Δ*Saro_RS13605*	86.706551	0.005999	0.420283	1.186903	0.525102	-4.960127
gMCS25	Δ*Saro_1894*Δ*pyk*Δ*Saro_RS09250*Δ*Saro_RS13605*	99.771405	0.005397	0.519385	0.598822	0.545712	-4.148006
gMCS26	Δ*Saro_2568*Δ*pyk*Δ*Saro_2679*Δ*Saro_0559*	101.833192	0.005190	0.670055	0.391970	0.566287	-3.859928
gMCS27	Δ*zwf*Δ*pyk*Δ*Saro_2679*Δ*Saro_RS09250*	113.764834	0.005228	0.769015	0.368973	0.566472	-1.744008
gMCS28	Δ*zwf*Δ*pyk*Δ*Saro_RS09250*Δ*Saro_2259*	90.088193	0.006157	0.533498	0.463802	0.520048	-4.384444
gMCS29	Δ*zwf*Δ*pyk*Δ*Saro_2679*Δ*Saro_0559*	96.727797	0.005218	0.503811	0.807930	0.520115	-3.192147
gMCS30	Δ*Saro_1894*Δ*pyk*Δ*Saro_2679*Δ*Saro_0559*	94.680007	0.005628	0.632000	0.556112	0.516318	-3.793410

Table 5 Knockouts for glycerate from vanillic acid. $Y_{x/s}$ (biomass yield, mgDW/mmol): biomass produced relative to substrate consumed. $Y_{p/x}$ (product yield, mmol/mgDW): product formed in relation to biomass produced. Min $Y_{p/s}$/max x (mmol/mmol): minimum product yield at maximum growth rate. Max $Y_{p/s}$/max x (mmol/mmol): maximum product yield at maximum growth rate. Min $Y_{p/s}$ (mmol/mmol):minimum product yield. %O2: oxygen ratio increasing (positive sign) or decreasing (negative sign) relative to wild-type. The name of the strains corresponds to the uppercase delta symbol followed by the name of the gene to be deleted.

Name	Strain	$Y_{x/s}$	$Y_{p/x}$	min $Y_{p/s}$ max x	max $Y_{p/s}$ max x	min $Y_{p/s}$	%O2
WT	WT	128.214809	0.000000	0.000000	0.105931	0.000000	
gMCS1	Δ*edd*Δ*pyk*Δ*Saro_RS09250*Δ*Saro_RS13605*	84.945501	0.006665	0.238496	1.538910	0.612860	-7.182241
gMCS2	Δ*edd*Δ*pyk*Δ*Saro_2679*Δ*Saro_RS09250*	70.835329	0.008639	0.644727	0.639201	0.612162	-10.034408
gMCS3	Δ*Saro_1894*Δ*pyk*Δ*Saro_RS09250*Δ*Saro_2259*	96.423541	0.005735	0.543836	0.707742	0.608855	-5.065019
gMCS4	Δ*Saro_1894*Δ*pyk*Δ*Saro_2679*Δ*Saro_RS09250*	73.366454	0.008107	0.575776	0.857203	0.603652	-9.376250
gMCS5	Δ*zwf*Δ*pyk*Δ*Saro_RS09250*Δ*Saro_RS13605*	95.252222	0.005923	0.746609	0.746609	0.655585	-6.526023
gMCS6	Δ*edd*Δ*pyk*Δ*Saro_RS09250*Δ*Saro_2259*	70.736439	0.008704	0.563686	0.994499	0.598431	-10.056422
gMCS7	Δ*edd*Δ*pyk*Δ*Saro_0559*Δ*Saro_2259*	72.929944	0.008291	0.564282	0.841001	0.593806	-9.582145
gMCS8	Δ*edd*Δ*pyk*Δ*Saro_2679*Δ*Saro_0559*	72.850545	0.008273	0.238496	1.538910	0.602683	-9.568122
gMCS9	Δ*Saro_2568*Δ*pyk*Δ*Saro_0559*Δ*Saro_2259*	78.943238	0.007645	0.659611	0.659611	0.612286	-8.689319
gMCS10	Δ*edd*Δ*pyk*Δ*Saro_0559*Δ*Saro_RS13605*	79.589506	0.007327	0.627106	0.650579	0.620983	-8.428183
gMCS11	Δ*pyk*Δ*zwf*Δ*Saro_0559*Δ*Saro_2259*	84.356211	0.007235	1.111129	1.111129	0.654142	-8.055825
gMCS12	Δ*Saro_2568*Δ*pyk*Δ*Saro_RS09250*Δ*Saro_2259*	82.381100	0.006697	0.673002	0.673002	0.612198	-7.360509
gMCS13	Δ*Saro_2568*Δ*pyk*Δ*Saro_2679*Δ*Saro_RS09250*	79.977757	0.007308	0.238496	1.538910	0.608844	-8.431750
gMCS14	Δ*Saro_1894*Δ*pyk*Δ*Saro_0559*Δ*Saro_RS13605*	72.760146	0.008276	0.238496	1.538910	0.607638	-9.543404
gMCS15	Δ*Saro_2568*Δ*pyk*Δ*Saro_RS09250*Δ*Saro_RS13605*	96.010850	0.005642	0.667538	0.667538	0.608860	-5.060201
gMCS16	Δ*pyk*Δ*zwf*Δ*Saro_0559*Δ*Saro_RS13605*	96.148665	0.005299	0.737490	0.737490	0.607907	-4.767246
gMCS17	Δ*Saro_1894*Δ*pyk*Δ*Saro_0559*Δ*Saro_2259*	99.424305	0.005332	0.657395	0.657395	0.608992	-4.640443
gMCS18	Δ*Saro_2568*Δ*pyk*Δ*Saro_0559*Δ*Saro_RS13605*	83.785020	0.007311	0.635189	0.994335	0.623293	-8.107449
gMCS19	Δ*Saro_1894*Δ*pyk*Δ*Saro_RS09250*Δ*Saro_RS13605*	82.299441	0.007522	0.795220	0.883288	0.657568	-8.478929
gMCS20	Δ*Saro_2568*Δ*pyk*Δ*Saro_2679*Δ*Saro_0559*	83.097740	0.006898	0.609788	0.810018	0.593810	-7.153749
gMCS21	Δ*zwf*Δ*pyk*Δ*Saro_2679*Δ*Saro_RS09250*	72.068631	0.008422	0.565885	0.943960	0.598492	-9.729417
gMCS22	Δ*zwf*Δ*pyk*Δ*Saro_RS09250*Δ*Saro_2259*	81.833995	0.007402	0.552057	0.672985	0.592490	-7.452928
gMCS23	Δ*zwf*Δ*pyk*Δ*Saro_2679*Δ*Saro_0559*	70.870377	0.008588	0.592880	0.756818	0.615279	-9.843727
gMCS24	Δ*Saro_1894*Δ*pyk*Δ*Saro_2679*Δ*Saro_0559*	89.920006	0.006596	0.238496	1.538910	0.630811	-6.962738

Table 6 Knockouts for phenol from vanillic acid. $Y_{x/s}$ (biomass yield, mgDW/mmol): biomass produced relative to substrate consumed. $Y_{p/x}$ (product yield, mmol/mgDW): product formed in relation to biomass produced. Min $Y_{p/s}$/max x (mmol/mmol): minimum product yield at maximum growth rate. Max $Y_{p/s}$/max x (mmol/mmol): maximum product yield at maximum growth rate. Min $Y_{p/s}$ (mmol/mmol):minimum product yield. %O2: oxygen ratio increasing (positive sign) or decreasing (negative sign) relative to wild-type. The name of the strains corresponds to the uppercase delta symbol followed by the name of the gene to be deleted.

Name	Strain	$Y_{x/s}$	$Y_{p/x}$	min $Y_{p/s}$ max x	max $Y_{p/s}$ max x	min $Y_{p/s}$	%O2
WT	WT	127.162223	0.000000	0.000000	0.080613	0.000000	
gMCS1	ΔeddΔpykΔSaro_2679ΔSaro_0559	119.485999	0.001142	0.195937	0.195937	0.160021	-2.339371
gMCS2	ΔeddΔpykΔSaro_RS09250ΔSaro_2259	103.964266	0.001175	0.132323	0.230804	0.141249	-3.589300
gMCS3	ΔeddΔpykΔSaro_RS09250ΔSaro_RS13605	106.149334	0.001205	0.192367	0.114377	0.146219	-2.647015
gMCS4	ΔzwfΔpykΔSaro_RS09250ΔSaro_2259	114.613473	0.001288	0.188769	0.082281	0.143638	-0.957710
gMCS5	ΔpykΔzwfΔSaro_2679ΔSaro_0559	119.277845	0.001155	0.191721	0.191721	0.158788	-1.531620
gMCS6	ΔSaro_1894ΔpykΔSaro_0559ΔSaro_RS13605	114.187434	0.001321	0.217206	0.087427	0.160688	-3.185552
gMCS7	ΔSaro_1894ΔpykΔSaro_RS09250ΔSaro_2679	95.535557	0.001611	0.155720	0.270823	0.147674	-6.265531
gMCS8	ΔeddΔpykΔSaro_0559ΔSaro_2259	107.984818	0.001297	0.150079	0.166513	0.146205	-3.154356
gMCS9	ΔSaro_2568ΔpykΔSaro_RS09250ΔSaro_2259	95.430278	0.001517	0.113373	0.188971	0.140537	-5.280391
gMCS10	ΔSaro_2568ΔpykΔSaro_0559ΔSaro_RS13605	93.212840	0.001571	0.143966	0.271020	0.148514	-5.593714
gMCS11	ΔzwfΔpykΔSaro_RS09250ΔSaro_2679	110.316309	0.001393	0.167626	0.101141	0.141704	-2.757328
gMCS12	ΔSaro_2568ΔpykΔSaro_0559ΔSaro_2259	105.678787	0.001375	0.193114	0.083970	0.148814	-3.874161
gMCS13	ΔSaro_1894ΔpykΔSaro_0559ΔSaro_2259	100.824321	0.001587	0.159968	0.247441	0.159968	-5.894558
gMCS14	ΔSaro_2568ΔpykΔSaro_RS09250ΔSaro_2679	92.085044	0.001587	0.135012	0.277483	0.147176	-5.657158
gMCS15	ΔzwfΔpykΔSaro_RS09250ΔSaro_RS13605	95.320523	0.001528	0.149263	0.263077	0.148570	-6.266701
gMCS16	ΔeddΔpykΔSaro_RS09250ΔSaro_2679	104.884141	0.001294	0.155911	0.155911	0.144672	-4.770534
gMCS17	ΔSaro_2568ΔpykΔSaro_RS09250ΔSaro_RS13605	123.335761	0.001177	0.180010	0.180010	0.146208	-0.628852
gMCS18	ΔeddΔpykΔSaro_0559ΔSaro_RS13605	115.870114	0.001058	3.018327	3.018327	0.147801	-1.114820
gMCS19	ΔpykΔzwfΔSaro_0559ΔSaro_2259	93.002693	0.001555	0.144665	0.213650	0.144665	-5.474694
gMCS20	ΔSaro_1894ΔpykΔSaro_RS09250ΔSaro_RS13605	95.128135	0.001517	0.144345	0.279829	0.144360	-5.273795
gMCS21	ΔSaro_1894ΔpykΔSaro_RS09250ΔSaro_2259	114.518947	0.001414	0.207080	0.174651	0.160435	-3.184496
gMCS22	ΔSaro_2568ΔpykΔSaro_2679ΔSaro_0559	93.063504	0.001561	0.080410	0.287001	0.142427	-5.502059
gMCS23	ΔSaro_1894ΔpykΔSaro_2679ΔSaro_0559	92.582410	0.001583	0.144942	0.290362	0.146198	-5.656948
gMCS24	ΔpykΔzwfΔSaro_0559ΔSaro_RS13605	104.397325	0.001519	0.158524	0.233951	0.158559	-5.328470

2 Candidate overexpression genes for the production of different bioproducts

The expression of a single gene can vary within a cell in response to different environmental factors or adaptation to a new food source. Different transcription factors often cause this variation.

In genetic engineering, overexpression of a gene refers to increasing its expression to enhance the flow toward a desired reaction. However, in a GEM, only two states of the gene - fully activated or completely inactive - are considered, and intermediate activation levels are ignored. Therefore, it is impossible to adjust a gene's expression to test if it leads to an increase in bioproduct synthesis. One way to tackle this issue is to identify genes that can be overexpressed by adjusting reaction rates. The key is to decrease the maximum rate of each reaction by a certain proportion and then evaluate whether this reduction affects the synthesis of the desired bioproduct. If this approach yields positive results, it can be inferred that increasing the expression of the genes involved in these reactions would increase the flux towards the production of the bioproduct.

This approach has certain limitations. A reaction can become rate-limiting when it is reduced, but this does not guarantee that any benefit is gained when it is increased from its initial value. Therefore, a number of reactions and associated genes that are involved in bioproduct formation and whose amplification could increase their accumulation are proposed here.

Table 7 Candidate overexpression genes for glutarate together with the name of the reaction they control. Reactions are named according to KEGG REACTION Database.

Gene name	Reaction name
Saro_2814, *ligK*, *Saro_2819*	OMA degradationase (LigK)
Saro_2819	PDC to OMA (LigI)
Saro_2811	CHMS_hemiacetal to PDC (LigC)
ligA, *Saro_2812*	PCA ring opening (LigAB)
Saro_2861	Vanillic acid demethylation (LigM)
Saro_2337, *lysA*	meso-2,6-diaminoheptanedioate carboxy-lyase
aspartate kinase	ATP:L-aspartate 4-phosphotransferase
folD	5,10-Methenyltetrahydrofolate 5-hydrolase
asd	L-Aspartate-4-semialdehyde:NADP+ oxidoreductase
dapE	N-Succinyl-LL-2,6-diaminoheptanedioate amidohydrolase
dapF	LL-2,6-Diaminoheptanedioate 2-epimerase
dapB	2,3,4,5-tetrahydrodipicolinate:NAD+ 4-oxidoreductase
dapD	Tetrahydrodipicolinate N-succinyltransferase
argD	Acetylornithine transaminase
dapA, *Saro_3897*	L-aspartate-4-semialdehyde hydro-lyase

Table 8 Candidate overexpression genes for citrate together with the name of the reaction they control. Reactions are named according to KEGG REACTION Database.

Gene name	Reaction name
Saro_2814, *ligK*, *Saro_2819*	OMA degradationase (LigK)
Saro_2819	PDC to OMA (LigI)
Saro_2811	CHMS_hemiacetal to PDC (LigC)
ligA, *Saro_2812*	PCA ring opening (LigAB)
Saro_2861	Vanillic acid demethylation (LigM)
Saro_2032	citrate oxaloacetate-lyase

Table 9 Candidate overexpression genes for propanoate together with the name of the reaction they control. Reactions are named according to KEGG REACTION Database.

Gene name	Reaction name
Saro_2814, *ligK*, *Saro_2819*	OMA degradationase (LigK)
Saro_2819	PDC to OMA (LigI)
Saro_2811	CHMS_hemiacetal to PDC (LigC)
ligA, *Saro_2812*	PCA ring opening (LigAB)
pobA	PHB hydroxyl attachment
Saro_1100	L-serine ammonia-lyase
aspartate kinase	ATP:L-aspartate 4-phosphotransferase
acs, *prpE*	Propanoate:CoA ligase AMP-forming
Saro_0867	acetyl-CoA:propanoyl-CoA 2-C-acetyltransferase
glyA	Serine hydroxymethyltransferase.
folD	5,10-Methenyltetrahydrofolate 5-hydrolase
Saro_0019	L-Homoserine:NAD+ oxidoreductase
asd	L-Aspartate-4-semialdehyde:NADP+ oxidoreductase
Saro_2511, *Saro_1705*, *Saro_0858*	S-2-methylbutanoyl-CoA:acceptor 2,3-oxidoreductase
Saro_1974	enzyme N6-(dihydrolipoyl)lysine S-(2-methylbutanoyl)transferase
ilvC	S-2-Aceto-2-hydroxybutanoate:NADP+ oxidoreductase
ilvD	R-2,3-Dihydroxy-3-methylpentanoate hydro-lyase
lpdA	Reaction lipoyllysine 2-oxidoreductase (decarboxylating)
ilvN, *ilvB*	pyruvate:2-oxobutanoate acetaldehydetransferase

Table 10 Candidate overexpression genes for phenol together with the name of the reaction they control. Reactions are named according to KEGG REACTION Database.

Gene name	Reaction name
tpiA	triose-phosphate isomerase
rpiB	D-ribose-5-phosphate aldose-ketose-isomerase
Saro_1964	beta-D-fructose-1-6-bisphosphate
rpe	D-Ribulose-5-phosphate 3-epimerase
tkt	transketolase
aroC	3-Phosphoshikimate 1-carboxyvinyltransferase
Saro_1352	Chorismate pyruvatemutase
Saro_0991	3-deoxy-7-phosphoheptulonate synthase
aroK	ATP:shikimate 3-phosphotransferase
aroE	Shikimate:NADP+ 3-oxidoreductase
aroB	3-dehydroquinate synthase
aroQ	3-Dehydroquinate hydro-lyase
aroA	3-phosphoshikimate 1-carboxyvinyltransferase
glpX	fructose-bisphosphatase class II

Table 11 Candidate overexpression genes for acetaldehyde together with the name of the reaction they control. Reactions are named according to KEGG REACTION Database.

Gene name	Reaction name
coaE	ATP:dephospho-CoA 3'-phosphotransferase
pyk, *ndk*	ATP:GDP phosphotransferase
ald	L-alanine:NAD+ oxidoreductase deaminating
cysN	ATP:adenylylsulfate 3'-phosphotransferase
cysN, *cysD*	ATP:sulfate adenylyltransferase
Saro_2558	hydrogen-sulfide:NADP+ oxidoreductase
rpiB	D-ribose-5-phosphate aldose-ketose-isomerase
purF	diphosphate phospho-alpha-D-ribosyltransferase (glutamate-amidating)
Saro_1309	1-(5'-Phosphoribosyl)-5-amino-4-(N-succinocarboxamide)-imidazole AMP-lyase
purH	5'-phosphoribosyl-5-amino-4-imidazolecarboxamide formyltransferase
purA	IMP:L-aspartate ligase GDP-forming
panB	3-methyl-2-oxobutanoate hydroxymethyltransferase
rpe	D-Ribulose-5-phosphate 3-epimerase
tkt	transketolase (tkt)
cysH	phosphoadenylyl-sulfate reductase
Saro_3896, *Saro_3892*, *Saro_RS08405*	R-Pantoate:NADP+ 2-oxidoreductase
panC	R-Pantoate:beta-alanine ligase AMP-forming
coaX	ATP:pantothenate 4'-phosphotransferase
coaD	ATP:pantetheine-4'-phosphate adenylyltransferase
coaBC	R-4'-phosphopantothenate:L-cysteine ligase
purD	phosphoribosylamine–glycine ligase
purM	phosphoribosylformylglycinamidine cyclo-ligase
purN	phosphoribosylglycinamide formyltransferase
purL, *purS*, *purQ*	Phosphoribosyl-N-formylglycinamide
purC	phosphoribosylaminoimidazolesuccinocarboxamide synthase
purK	protein=5-(carboxyamino)imidazole ribonucleotide synthase
purE	protein=5-(carboxyamino)imidazole ribonucleotide mutase

Table 12 Candidate overexpression genes for glycerol together with the name of the reaction they control. Reactions are named according to KEGG REACTION Database.

Gene name	Reaction name
Saro_0085, *Saro_2442*, *gpsA*	sn-Glycerol-3-phosphate:NAD+ 2-oxidoreductase

Table 13 Candidate overexpression genes for 1-hexadecanol together with the name of the reaction they control. Reactions are named according to KEGG REACTION Database.

Gene name	Reaction name
folD	5,10-Methenyltetrahydrofolate 5-hydrolase
fabZ	(3R)-hydroxymyristoyl acyl carrier protein dehydrase
Saro_3306, *fabI*	hexadecanoyl-[acp]:NAD+ trans-2-oxidoreductase
fabG	3-oxoacyl-[acyl-carrier-protein] reductase
fabF	malonyl-[acyl-carrier-protein] C-acyltransferase
gpsA, *Saro_2617*	carbonate hydro-lyase carbon-dioxide-forming
fabH	malonyl-[acyl-carrier-protein] C-acyltransferase

3 Scoring of genetic interventions

We adopted those scoring criteria that best fit the characteristics and available information of our model. To obtain an overall quantitative measure Si for each intervention strategy i, an overall score is calculated from the individual scores $S_{i,j}$ for each of the ten criteria j = 1...10. The score for each criterion $S_{i,j}$ can take normalised values between 0 and 1. Intervention strategy i that takes the most unfavourable value on criterion j is scored $S_{i,j} = 0$ and strategy k with the most favourable value is scored $S_{k,j} = 1$.

Specifically, if a high value $U_{i,j}$ of a specific criterion j (e.g. minimum product yield) is desirable, the score of strategy i is determined by of strategy i is determined by:

$$S_{i,j} = \frac{U_{i,j} \cdot U_{j,\min}}{U_{j,\max} \cdot U_{j,\min}} \tag{1}$$

In the case of a preferably low value $U_{i,j}$, the term is:

$$S_{i,j} = \frac{U_{j,\max} \cdot U_{i,j}}{U_{j,\max} \cdot U_{j,\min}} \tag{2}$$

Criteria with a preferably low value are the number of interventions, oxygen consumption and the number of accessible metabolites. The total score S_i for an intervention strategy i is then a weighted sum of its scores for the criteria:

$$S_i = \sum_j S_{i,j} \tag{3}$$

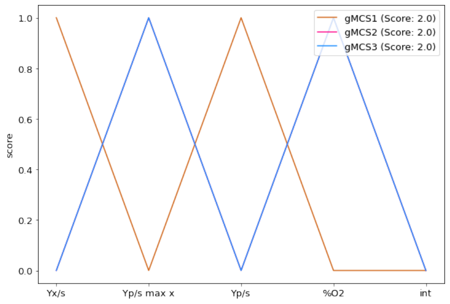

(a) Glutarate

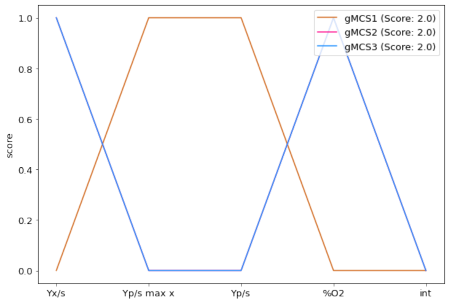

(b) Citrate

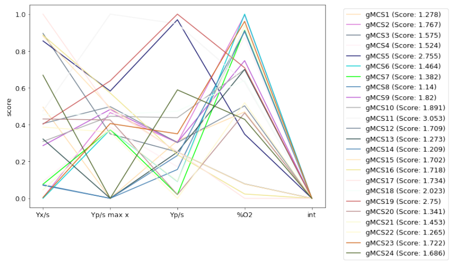

(c) Glycerol

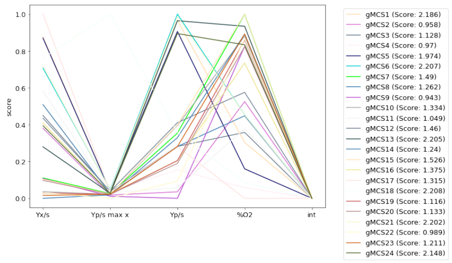

(d) Phenol

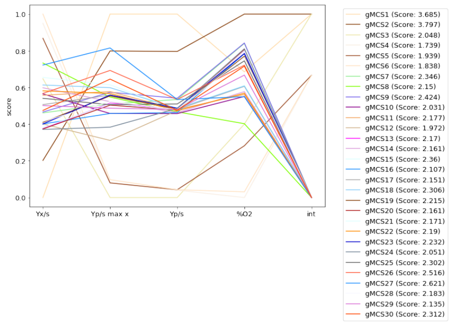

(e) Acetaldehyde

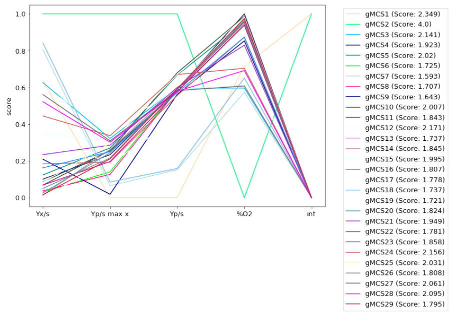

(f) Propianoate

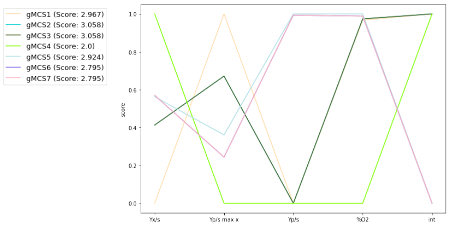

(g) PDC

Figure 1: Scoring the intervention strategies for bioproducts.
